# Background correction using dinucleotide affinities improves the performance of GCRMA

**DOI:** 10.1186/1471-2105-9-452

**Published:** 2008-10-23

**Authors:** Raad Z Gharaibeh, Anthony A Fodor, Cynthia J Gibas

**Affiliations:** 1 Department of Bioinformatics and Genomics, University of North Carolina at Charlotte, Charlotte, NC, 28223, USA

## Abstract

**Background:**

High-density short oligonucleotide microarrays are a primary research tool for assessing global gene expression. Background noise on microarrays comprises a significant portion of the measured raw data, which can have serious implications for the interpretation of the generated data if not estimated correctly.

**Results:**

We introduce an approach to calculate probe affinity based on sequence composition, incorporating nearest-neighbor (NN) information. Our model uses position-specific dinucleotide information, instead of the original single nucleotide approach, and adds up to 10% to the total variance explained (R^2^) when compared to the previously published model. We demonstrate that correcting for background noise using this approach enhances the performance of the GCRMA preprocessing algorithm when applied to control datasets, especially for detecting low intensity targets.

**Conclusion:**

Modifying the previously published position-dependent affinity model to incorporate dinucleotide information significantly improves the performance of the model. The dinucleotide affinity model enhances the detection of differentially expressed genes when implemented as a background correction procedure in GeneChip preprocessing algorithms. This is conceptually consistent with physical models of binding affinity, which depend on the nearest-neighbor stacking interactions in addition to base-pairing.

## Background

Affymetrix GeneChip arrays are one of the most popular gene expression array systems used by researchers worldwide [1]. The purpose of an expression microarray experiment is to measure the abundance of each known transcript in the sample under investigation. Abundance is inferred from the signal generated by a set of 11–20 probe pairs. Each pair is composed of a perfect match probe (PM), which exactly complements a region on the transcript, and a mismatch probe (MM), which is identical to the PM probe except at the 13^th ^base, where the reverse complement nucleotide is introduced [2]. The fluorescent signal from each probe, however, includes *background noise *that not only measures the transcript abundance, but also non-specific binding (NSB) and autofluorescence of the chip surface. MM probes were originally introduced by Affymetrix to measure background noise. It has been shown by many groups that MM probes contain significant amount of the PM signal and are therefore unreliable as estimators of background noise [3-5].

A gene expression experiment using the Affymetrix GeneChip system usually involves a design step, a preprocessing step, an inference step and finally, a validation step [6]. The preprocessing step is of special importance; preprocessing transforms the raw fluorescence signals from each probe in a probeset into a composite gene expression value. The main goal of the preprocessing step is to remove non-biological variation from the raw data [6]. Usually, the preprocessing step in Affymetrix GeneChip array analysis includes three main treatments of the raw data. A background adjustment step separates the specific signal from the non-specific signal. A probe-level normalization step then removes non-biological variation between arrays. Finally, a summarization step generates a single expression value for each gene from its corresponding probeset. The method described in this manuscript is an implicit physical model that modifies the background adjustment step.

Background noise and non-biological variation of the signal generated from each probe are common phenomena in GeneChip microarray experiments [7,8]. The differences in the signal produced can be attributed to many sources: optical noise, cross-hybridization, dye-related contributions and probe sequence composition. Many preprocessing algorithms have been developed in an attempt to correct for these artifacts [9]. According to Allison et al. [6] there is no clear winner among the available preprocessing algorithms. However, GCRMA [10], a modification of RMA [11], often performs as well as or better than other algorithms [9,12-14]. GCRMA incorporates probe sequence composition into background adjustment, following the physical model of Naef and Magnasco [15]. The model describes a probe affinity that is dependent on its base composition and the position of each base along the probe and suggests that probe sequence can significantly affect the intensity of the signal generated from that probe, independent of the concentration of its target.

Performance assessment of GCRMA has been done using both spike-in [13,16,17] and real [14] datasets followed by quantitative real time PCR confirmation [12]. So far, a number of reports have been published recommending the use of GCRMA for detecting differentially expressed genes and estimating relative expression, emphasizing its outstanding performance in detecting low-intensity, differentially expressed genes [13,17]. When comparing microarray analysis algorithms, Irizarry et al. [9] have argued for an approach that balances accuracy and precision. Irizarry et al., define *accuracy *as the ability of the algorithm to detect the relative expression of a transcript without bias to its abundance (concentration). They define *precision *as low variance; this is characterized by a steady performance on replicates of the same sample. GCRMA is among the few preprocessing algorithms that scores well in both accuracy and precision [13].

In this study, we modified the portion of GCRMA derived from the model of Naef and Magnasco [15] to calculate probe affinity using position-specific dinucleotide information. The dinucleotide is a fundamental chemical unit that contributes a well-understood component to nucleic acid duplex stability and to the free energy of duplex formation during hybridization [18,19]. We applied the new model to different datasets, and achieved an improved fit to microarray data with R^2 ^increasing by 5–10%. Then, we tested the downstream effect of our modified background model on the performance of GCRMA in detecting differentially expressed genes, when used to analyze two publicly available control datasets: the human genome U133 Latin Square dataset [20] and the golden spikein dataset [16]. In both data sets, application of the dinucleotide model in background correction improved the detection of differentially expressed genes. Therefore, we propose that probe affinity be modeled based on dinucleotide composition of the probe instead of the original single nucleotide approach.

## Results

### Dinucleotide affinity model

Naef and Magnasco [15] model *probe affinity *(probe hybridization effect) based on sequence composition as follows:

(1) $$\ln\langle B/M\rangle =\sum_{k=1}^{25}\sum_{l\in (A,T,C,G)}S_{lk}A_{lk}$$

where *B *is the raw probe intensity, *M *is the median intensity of the array, *l *is the nucleotide index (A, C, G or T), *k *is the position of *l *along the probe (note that *k *has a range of 1 to sequence length, that is 25 for GeneChip probes), *S *is a Boolean variable equal to 1 if the probe sequence has *l *at *k *and zero otherwise, and *A *is the per-site-per- nucleotide affinity. As an example, consider the following sequence: CGAC, for which equation 1 reads:

$$\begin{array}{l} \ln\langle B/M\rangle =\\ \left(S_{1G}\times A_{1G}\right)+\left(S_{1A}\times A_{1A}\right)+\left(S_{1T}\times A_{1T}\right)+\left(S_{1C}\times A_{1C}\right)+\\ \left(S_{2G}\times A_{2G}\right)+\left(S_{2A}\times A_{2A}\right)+\left(S_{2T}\times A_{2T}\right)+\left(S_{2C}\times A_{2C}\right)+\\ \left(S_{3G}\times A_{3G}\right)+\left(S_{3A}\times A_{3A}\right)+\left(S_{3T}\times A_{3T}\right)+\left(S_{3C}\times A_{3C}\right)+\\ \left(S_{4G}\times A_{4G}\right)+\left(S_{4A}\times A_{4A}\right)+\left(S_{4T}\times A_{4T}\right)+\left(S_{4C}\times A_{4C}\right)\\ \ln\langle B/M\rangle =A_{1C}+A_{2G}+A_{3A}+A_{4C} \end{array}$$

Equation 1 is a simple model that has four free parameters for each probe base (100 free parameters for a 25-base probe). The values of these 100 free parameters are generated by linear least squares fit. Given the large number of probes on each chip (about half a million for the human genome U133 chip, for example) over-fitting is not a concern.

**Figure 1:**
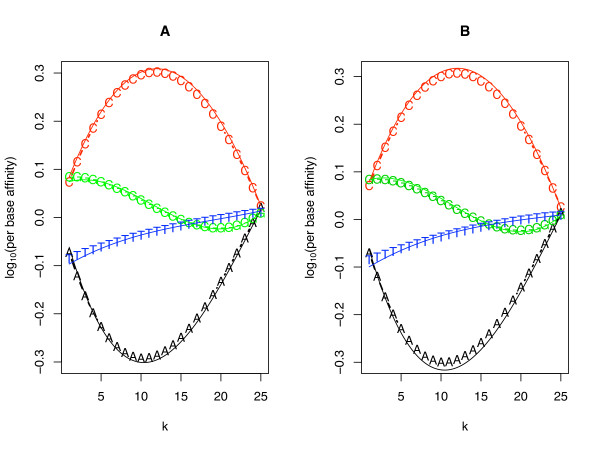
**Affinity parameters calculated using single nucleotide model**. Affinity parameters calculated using equation 1 for the human genome U133 Latin Square. Panel A is for Experiment 11 Replica 2 and panel B shows an average of the parameters across all the 42 chips. K represents the position of each nucleotide along the probe length. Affinity parameters calculated using equation 2 are shown as solid lines. Higher affinity (Y-axis) indicates brighter signal.

Figure 1 shows the 25 parameters (term *A *in equation 1) of the four nucleotides as a function of their position along the probe for the U133 Latin square dataset (parameters derived from a single chip are shown in panel A and an average of the parameters across all the 42 chips is shown in panel B). A similar pattern of parameters have been obtained fitting equation 1 to other Affymetrix datasets (data not shown and [15]). These fitted per-site-per-nucleotide affinities imply that the signal generated from each probe will be affected by the probe sequence. Consider two probes interrogating two transcripts, which are present in identical concentration. In such a case, a probe containing many adenines (A) will produce a lower signal intensity than the probe with many cytosines (C), especially if the As or Cs are concentrated at or near the center of the probe (position 13).

The model defined in equation 1 can also be expressed as a polynomial of degree 3, thus reducing the free parameters from 100 to 16 as shown below:

(2) $$\ln\langle B/M\rangle =\sum_{k=1}^{25}\sum_{l\in (A,T,C,G)}\sum_{t=0}^{3}S_{lk}A_{lt}k^{t}$$

By assuming the affinities can be modeled as a third order polynomial function of position, the number of free parameters in the model can be reduced from 100 to 16 with little loss of predictive accuracy as the polynomial generated with 16 parameters (Fig. 1 solid lines) closely matches the 100 independently estimated parameters (Fig. 1 symbols) and the R^2 ^of both models are similar (additional file 1).

In the dinucleotide model, we follow a similar strategy to the above, but we model composition-biased probe affinity using dinucleotides (pairs of adjacent bases), which are a fundamental chemical unit in physical models of nucleic acid folding and hybridization rather than single nucleotides. The dinucleotide model is as follows:

(3) $$\ln\langle B/M\rangle =\sum_{k=1}^{25}\sum_{l\in NN}S_{lk}A_{lk}$$

where *B *is the raw probe intensity, *M *is the median intensity of the array, *l *is the NN nucleotide pair (AA, AC AG, AT, CA, CC, CG, CT, GA, GC, GG, GT, TA, TC, TG or TT), *k *is the position of *l *along the probe (note that *k *has a range of 1 to sequence length minus one, that is 24 for GeneChip probes), *S *is a Boolean variable equal to 1 if the probe sequence has *l *at *k *and zero otherwise, and *A *is the per-site-per-dinucleotide affinity. We then again assume that the per-site-per-dinucleotide affinity follows a polynomial of degree 3 as a function of the position *k *as outlined in equation 4:

(4) $$\ln\langle B/M\rangle =\sum_{k=1}^{25}\sum_{l\in NN}\sum_{t=0}^{3}S_{lk}A_{lt}k^{t}$$

This reduces the number of free parameters from 384 (16 dinucleotides × 24 nucleotide positions, equation 3) to 64 (16 dinucleotides × 4 parameters, equation 4), which makes this approach computationally feasible. As an example, consider the following sequence: CGAC (three dinucleotides: CG for *k *= 1, GA for *k *= 2, and AC for *k *= 3), for which equation 4 reads:

$$\begin{array}{l} \ln\langle B/M\rangle =\\ (A_{CG0})+(A_{CG1}\times 1)+(A_{CG2}\times 1^{2})+(A_{CG3}\times 1^{3})+\\ (A_{GA0})+(A_{GA1}\times 2)+(A_{GA2}\times 2^{2})+(A_{GA3}\times 2^{3})+\\ (A_{AC0})+(A_{AC1}\times 3)+(A_{AC2}\times 3^{2})+(A_{AC3}\times 3^{3})+\\ \ln\langle B/M\rangle =4A_{CG}+15A_{GA}+40A_{AC} \end{array}$$

Note that we do not explicitly fit the stacking energies of the NN pairs; rather we explicitly fit the NN pairs' affinities along the probe sequence position.

**Figure 2:**
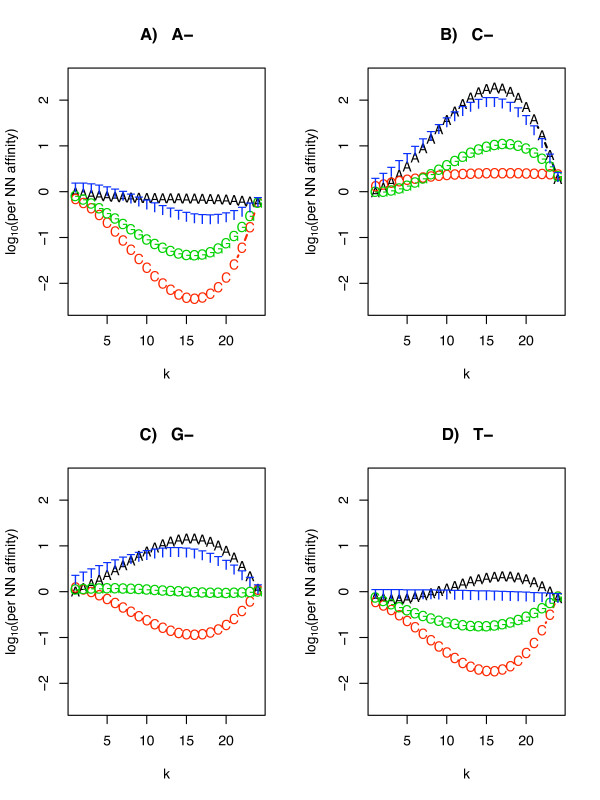
**Affinity parameters calculated using dinucleotide model**. Affinity parameters calculated using equation 4 for the human genome U133 Latin Square. Affinity parameters are averaged across all the 42 chips; parameters for any single chip resemble those shown here. The first letter of each dinucleotide is indicated at the top of the figure, the second letter is indicated on the connected lines. K represents the position of each dinucleotide along the probe length. Higher affinity (Y-axis) indicates brighter signal.

The fitted per-site-per-dinucleotide affinities are shown in Fig. 2 for the Latin square dataset. Parameters obtained from other datasets are similar to the Latin square dataset parameters (data not shown). The figure shows that a probe with many AN (N = A, C, G, T) pairs (Fig 2A) tends to have much lower intensity than a probe with many CN pairs (Fig 2B) especially when those pairs are located at or near the probe center. This is broadly what we expect from the single nucleotide model. However, examining the effect caused by second nucleotide in each NN pair shows a pronounced effect for certain dinucleotides, which cannot be captured in the single nucleotide model. This can be seen in Fig. 2C and 2D. GA and GT rich probes are significantly brighter than GC rich probes, and TA rich probes are brighter than TC and TG rich probes.

The model defined in equation 4 was fitted to a number of datasets (see Methods). Fitting was performed on the PM and MM probes separately. Table 1 shows a comparison between the native Naef and Magnasco [15] affinity model (single nucleotide model, equation 1) and our dinucleotide affinity model (equation 4). We see that the dinucleotide model gives a better fit to microarray data by 5–10% on average (Table 1 and additional file 1), depending on the chip and probe type. Note that both models perform better on the MM probes due to the higher background noise present in the MM signal.

**Table 1:** Dinucleotide model performance on different datasets

Data set	*nc*^a^	*np*^b^		Single nucleotide model (eq. 1)	Dinucleotide model (eq. 4)^c^
Latin Square [20]	42	248152	*PM*	0.17 ± 0.01	0.22 ± 0.01
		248152	*MM*	0.40 ± 0.01	0.50 ± 0.01
					
Golden spikein [16]	6	195994	*PM*	0.20 ± 0.02	0.22 ± 0.02
		195994	*MM*	0.46 ± 0.02	0.51 ± 0.02
					
Leukemia [35]	72	201800	*PM*	0.49 ± 0.06	0.55 ± 0.07
		201800	*MM*	0.60 ± 0.04	0.69 ± 0.04
					
Etoposide response [34]	60	496468	*PM*	0.05 ± 0.04	0.08 ± 0.06
					
		496468	*MM*	0.11 ± 0.06	0.16 ± 0.08
BK knockout [36,37]	20	496468	*PM*	0.09 ± 0.04	0.13 ± 0.04
		496468	*MM*	0.29 ± 0.050	0.36 ± 0.06

R2 of Naef and Magnasco [15] model (Single nucleotide) and the dinucleotide model for the five data sets used in this study. Results presented as average R2 ± SD.

^a^*nc*: number of chips.

^b^*np*: number of probes.

^c ^The differences in R^2 ^between single nucleotide model and dinucleotide model are all statistically significant (*p *< 10^-3^) using paired one-sided Wilcoxon and *t *tests.

Given that our fits contain between 195,994 and 496,468 data points (Table 1), it seems unlikely that the improvements in performance of our model could be explained by the additional free parameters (64 for our model vs. 16 for the original Naef and Magnasco model). Nonetheless, to rule out this possibility, we fitted both the single nucleotide model (N) (using the 100 free parameters and 16 free parameter version of the Naef and Magnasco model, equation 1 and 2, respectively) and the dinucleotide model (NN) with 64 free parameters (equation 4) to the Latin Square dataset using completely random probe sequences (generated with an equal probability of A, C, G and T). We also performed the same test on shuffled probe sequences in which the probe's base composition is not affected, but the position of each base has been changed due to the shuffling process. The results of this analysis are shown in additional file 1. We see that the R^2 ^of the shuffled and random probe sequences are nearly identical, no matter which method is used. The presence of additional free parameters in our model, therefore, cannot by itself explain the improved performance over the Naef and Magnasco model. This strongly supports our argument that the gain in the r-squared values of the NN model comes from including dinucleotide information and does not arise trivially from the addition of free parameters.

### Background adjustment using dinucleotide affinity model

Using a more accurate estimate of background noise should improve the quality of Affymetrix GeneChip data. Given the better fits observed using the dinucleotide affinity model, we expected it to improve the analysis results to some degree when applied to control datasets. We tested the downstream effects of using this model on the quality of microarray data. We chose to implement the model within GCRMA [10], since it already has the single nucleotide model implemented in its background correction procedure, and therefore the two models could be directly compared.

In GCRMA, Wu et al. [10] model the signal intensity generated from each probe as:

(5) $$\begin{array}{l} PM=O_{PM}+N_{PM}+S,\\ MM=O_{MM}+N_{MM}+\phi S \end{array}$$

where *O *is the optical noise, *N *is the background noise of non-specific binding, and *S *is the signal generated from specific binding between the probe and its intended target. The parameter φ reflects the fact that for some probe pairs, the MM signal may contain specific signal. The background components log(*N*_*PM*_) and log(*N*_*MM*_) are assumed to follow a bivariate distribution with means of μ_*pm *_= *h*(α_*PM*_) and μ_*mm *_= *h*(α_*MM*_), where *h *is a smoothing function and α (probe affinity) is defined by equation 1. In this paper, we make these same assumptions, but we derive α using equation 4.

We reasoned that GCRMA with background correction using the dinucleotide model, which we will subsequently refer to as GCRMA-NN in this paper, would perform better than the native GCRMA model. It is important to clarify that GCRMA offers two options for background correction, the first of which uses a precomputed α (called reference affinity) from the authors' own non-specific binding (NSB) experiments, while the second computes α directly from the data (called local affinity). In the following figures, we compare GCRMA-NN (where α is computed directly from the data using equation 4) to GCRMA-L (GCRMA with local affinity) and GCRMA-R (GCRMA with reference affinity).

### Latin square dataset

We obtained expression measures for the Human Genome U133 Latin square dataset after processing it with GCRMA-R, GCRMA-L and GCRMA-NN. The three expression measures were evaluated using two approaches. The first approach is based on AffyComp [21], a performance evaluation tool for preprocessing algorithms (see below). The second approach is based on the number of true positives captured for all the 14 2× comparisons of the Latin square dataset at a cutoff of four false positives after using the cyber *t *test [22]. Cyber *t *is a popular variant of the *t *test, in which a weighted standard deviation replaces the conventional standard deviation and an adjusted number of degrees of freedom is used instead of the conventional degrees of freedom.

Performance of GCRMA-R, GCRMA-L and GCRMA-NN as reported by AffyComp based on 14 metrics is shown in Table 2. One notable performance enhancement of GCRMA-NN over GCRMA-L and GCRMA-R is a 3–4% increase in the weighted average area under the curve (AUC) (Table 2). This is a receiver operator characteristics (ROC) based metric, in which the absolute log-ratios for the expression summaries, for every comparison of any two pairs of the 14 arrays (92 comparisons), are sorted. After that, the number of true and false positives is found, and then the number of true positives at 100 false positives is determined for each pair of arrays. Finally, the resulting values are averaged over the three concentration groups (low, med and high), weighted by the number of probesets in each group and a score is recorded. Note that a perfect algorithm will have a score of 1, where all the true positives are captured before any false positive is recorded.

**Table 2:** AffyComp scores for GCRMA-L, GCRMA-R and GCRMA-NN

Metric	GCRMA-L	GCRMA-R	GCRMA-NN	Perfect score
Median SD	0.06	0.06	0.07	0
null log-fc IQR	0.05	0.03	0.08	0
null log-fc 99.9%	0.62	0.61	0.64	0
Signal detect slope	0.99	1	0.98	1
Signal detect R^2^	0.89	0.91	0.91	1
low.slope	0.49	0.48	0.55	1
med.slope	1.05	1.06	1.02	1
high.slope	0.97	0.97	0.96	1
Obs-intended-fc slope	0.99	1	0.98	1
Obs-(low)int-fc slope	0.48	0.47	0.53	1
low AUC	0.44	0.45	0.50	1
med AUC	0.87	0.87	0.86	1
high AUC	0.85	0.86	0.83	1
weighted avg AUC	0.55	0.56	0.59	1

Fourteen AffyComp metrics for the U133 Latin square dataset rounded to two decimal points.

A brief description of each metric is provided under the Methods section.

Examining Table 2 shows that the increase comes mainly from the AUC for low intensity targets (low AUC entry in Table 2). The low intensity genes make up most of the genes in a typical Affymetrix experiment [13] and are also the hardest to detect. Algorithms that perform inference generally can detect large changes involving highly expressed genes. It is much more difficult to detect changes in the more frequently observed genes that produce low intensities on the array. GCRMA-NN enhanced the detection of low intensity targets, while maintaining similar values for the medium and high intensity ones. The enhancement in detecting low intensity targets is also evident in the form of an increase in the low detection slope (low.slope entry in Table 2).

**Figure 3:**
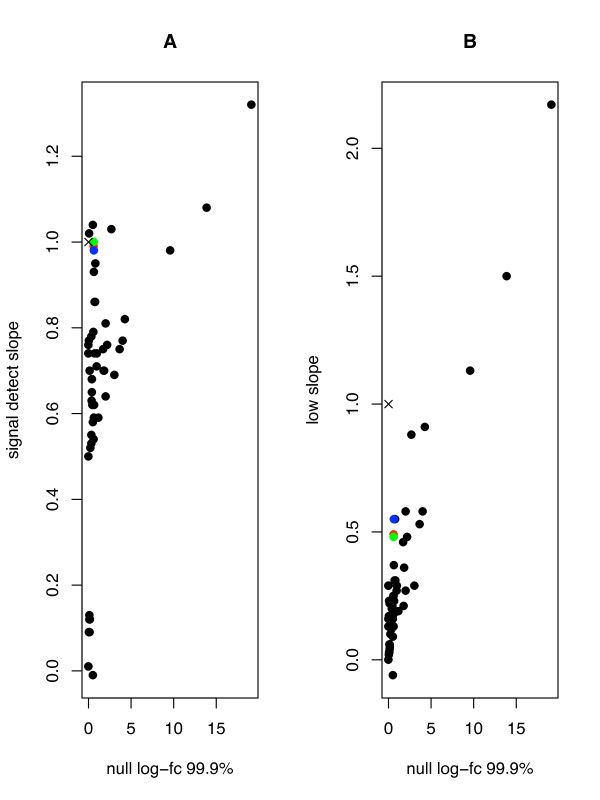
**GCRMA-NN accuracy and precision**. A) Accuracy and precision of GCRMA-R (green dot), GCRMA-L (red dot) and GCRMA-NN (blue dot) compared to other preprocessing algorithms (black dots) submitted to AffyComp [33], information retrieved from AffyComp on November, 14^th ^2007. B) As A but for low expressed genes. A perfect score is shown as an (×) on both panels. See Results for explanation.

In the crucial category of low intensity genes, we argue that our algorithm outperforms most of the algorithms submitted to AffyComp, including GCRMA-R and GCRMA-L. The AffyComp webpage currently contains data for 88 algorithms for analyzing Affymetrix microarrays. For each of these algorithms, AffyComp defines accuracy as the slope obtained from regressing expression values on nominal concentration. An algorithm with a perfect accuracy would have a slope of 1, reflecting a perfect correspondence between nucleotide concentration and signal. AffyComp defines precision as the 99.9% percentile of the log fold changes of null (true negative) probesets across arrays. A perfect algorithm would have a precision of 0 reflecting a fold change of 1 (i.e. no change). Figure 3 is a plot of precision vs. accuracy for the Latin Square dataset for the 88 algorithms submitted to the AffyComp webpage. In Figure 3A, we see that when looking at overall accuracy vs. precision, the GCRMA-NN algorithm (blue dot) performs about as well as GCRMA-R (green dot) and GCRMA-L (red dot). However, for the crucial low intensity genes, for which inference is the most difficult, GCRMA-NN provides a better accuracy with no loss of precision (Fig. 3B).

**Figure 4:**
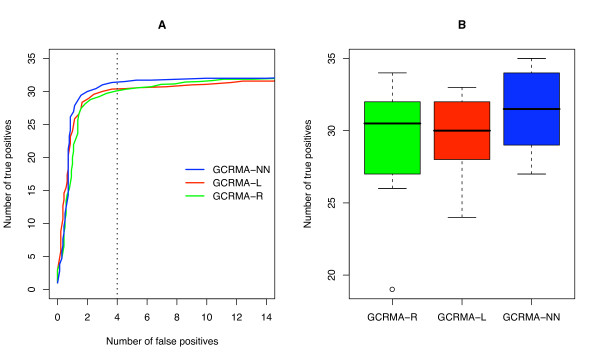
**Performance of GCRMA-R, GCRMA-L and GCRMA-NN on the Latin square dataset**. A) ROC curves showing the average true positives and false positives across the 14 2× Latin square experiments following application of the cyber *t *test. B) The number of true positives captured for all the 14 2× Latin square experiments at a cutoff value of four false positives (dashed vertical line in panel A). The differences in panel B between GCRMA-R, GCRMA-L and GCRMA-NN are statistically significant (*p *< 0.005) using paired one-sided Wilcoxon and *t *tests.

Since the results of AffyComp suggest an improvement for the low intensity, hard to detect spikeins, we reasoned that inference performed with GCRMA-NN would be more successful than inference with GCRMA-R or GCRMA-L. We therefore applied GCRMA-NN, GCRMA-R and GCRMA-L to the U133 Latin square dataset. We considered only the 14 2× comparisons, in which the ratio of each spikein, between any two consecutive pair of arrays, is 2. Then we used the cyber *t *statistic [22] to generate a list of *P *values for the null hypothesis that the mean signal intensity in each comparison is the same. The lists were ordered, and for each of the 14 comparisons we generated an ROC curve. Figure 4A shows the average of these 14 ROC curves. For each ROC curve, we determined the number of true positives captured at an arbitrary cutoff of four false positives (vertical dashed line in Fig. 4A). The result of this analysis is summarized in Figure 4B. We see that GCRMA-NN outperforms GCRMA-R and GCRMA-L with a small but significant improvement. One-sided Wilcoxon and *t *tests reject the null hypothesis that GCRMA-NN is the same as GCRMA-R and GCRMA-L with all tests *p *< 0.005. These are consistent with the results we would have expected based on the AffyComp comparison (Table 2).

### Golden spikein dataset

**Figure 5:**
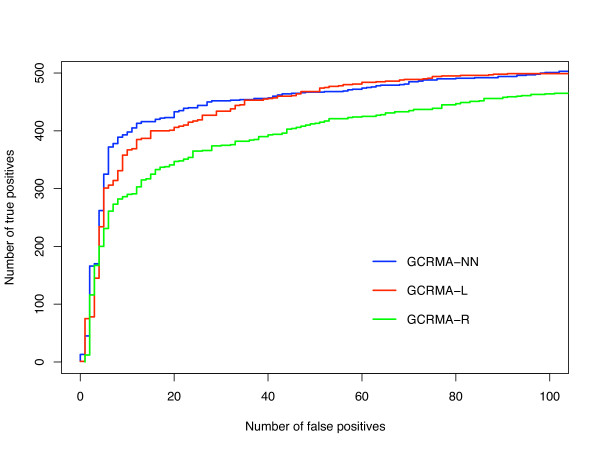
**Performance of GCRMA-R, GCRMA-L and GCRMA-NN on the Golden spikein dataset**. ROC curves for the Golden spikein experiments C versus S after application of the cyber *t *test.

In order to ensure that our data were valid for more than one control data set, we next applied GCRMA-R, GCRMA-L and GCRMA-NN to the "golden spikein dataset" [16], which is not included in AffyComp. Figure 5 shows a ROC graph for the differentially expressed genes between the S and the C "golden spike" samples (see Methods) detected by GCRMA-R, GCRMA-L and GCRMA-NN. As in the Latin Square data, the graph shows that GCRMA-NN is capable of capturing more true positives at lower false positive rate than both GCRMA-R and GCRMA-L. This supports our assertion that an improved background correction algorithm can have a noticeable effect on downstream analyses.

## Discussion

Background estimation and correction are important steps in analyzing the data generated by GeneChip arrays. Improving algorithms for these steps increases the amount of true "signal" that we can detect from microarrays. Understanding background noise on GeneChip arrays, especially the part contributed by NSB signal, requires a deeper understanding of the behavior of on-chip hybridization. Given that we lack a detailed physical model of on-chip hybridization derived from first principles, an empirical model that estimates the specific and non-specific signal based on the data on the array and probe sequence is a useful tool for understanding the on-chip hybridization process.

Nucleic acid hybridization in solution is well approximated by the nearest neighbor model [23], which describes duplex formation as a function of the two adjacent nucleotides and their stacking orientation. This approach was used by Zhang et al. [24] to model the on-chip specific and nonspecific hybridization using the free energy formation for the adjacent nucleotides. Zhang et al. concluded that the on-chip hybridization parameters are different than the solution ones. Using a different approach to background correction, Naef and Magnasco [15] used single nucleotides to assign an overall affinity score for a probe based on its sequence away from the energy contributions of the dinucleotide pairs. This approach was used to perform background correction for the GCMRA algorithm [10] while the Zhang et al approach was used to create the algorithm PerfectMatch [24]. PerfectMatch estimates the signal and the background at the same step while GCRMA estimates background noise first then proceed to signal estimation. PerfectMatch is, therefore, much more computationally demanding than GCRMA as the parameter space searched by PerfectMatch is vast and is sampled with Monte Carlo methods. Direct comparison of GCRMA and PerfectMatch has proven controversial. Such a comparison is beyond the scope of this report, and can be found elsewhere [9,13,25].

In this report we combine some elements of GCRMA and PerfectMatch. We replace the single nucleotide model of Naef and Magnasco with a model in which the affinity of each probe is a function of its *dinucleotide *composition. Because we use GCRMA's approach of separating estimates of background and signal, we can use a linear model and avoid the Monte Carlo simulation approach of PerfectMatch [24]. Our approach is therefore both computationally more efficient and guarantees the best fit to the data. This approach enables us to examine the contribution of different dinucleotides at different positions to the raw probe signal (Fig. 2), rather than assigning one weight function to all the dinucleotides, as is done with PerfectMatch [24]. This allows our model to capture several important features of the background data such as the effect of the first versus the second nucleotide on probe affinity (e.g. CA vs. CG), and the effect of the stacking orientation (AC vs. CA). In general, we find that the dinucleotide approach has more power than the single nucleotide approach over a wide range of datasets (Table 1).

The mechanism that determines why particular dinucleotides affect probe affinities the way they do is, in some cases, unclear. However, we observe that the NN model bears some similarities to the models of both Naef and Magnasco and Zhang et al. All three models emphasize the importance of the probe middle region; this is probably due to the surface attachment, as well as to the relative instability of the free end in RNA-DNA hybridization. The effect of the stacking orientation is in agreement with the findings of Zhang et al. [24]. The AN versus CN (where N refers to any of the four nucleotides: A, C, G, T; AN for example means AA, AC, AG and AT) asymmetry (Fig. 2A and 2B) is in agreement with Naef and Magnasco [15]. When comparing these affinity curves to the original Naef and Magnasco result, it is important to recognize that the NN model considers the affinity of dinucleotides rather than single nucleotides. Therefore, we do not necessarily expect to see the same asymmetry within CN or AN, i.e. there will be no asymmetry between CA and CC (Fig. 2B), or between AA and AC (Fig. 2A). The NN model, however, does show unexpected behavior for the GN and TN dinucleotides. While both G and T show slight asymmetry in the Naef and Magnasco model, the effect of these two nucleotides is magnified in the NN model. GN contributes positively to the signal but not when the second nucleotide is C (Fig. 2C). TN contributes negatively but not when the second nucleotide is A (Fig. 2D). This trend is partially explained by the fact that T forms fewer hydrogen bonds than G, therefore contributing negatively, while the G has stronger binding, thus contributing positively. This trend is not consistent, and appears to be dependent on the adjacent nucleotide. It could also be due to the biotin label present on the RNA target sequence.

When applied to two control datasets, GCRMA-NN showed improved performance (Figs. 4, 5) especially on low intensity targets (Table 2; Fig. 3). We argue that this is due to better background correction for these targets; a higher percentage of low intensity signal will be made up of background, so it is therefore not surprising that better background correction will make more of a difference on low intensity targets. The detection of low intensity targets represent the most significant challenge to microarray analysis algorithms, which makes any enhancement in the detection of these targets significant.

## Conclusion

Incorporating dinucleotide information into a previously described probe affinity model increases the fit of the model by 5–10%. The dinucleotide affinities highlight the importance of the stacking orientation on probe behavior. This is in agreement with the physical models that describe hybridization binding affinities.

The results presented here show that the affinity of any single nucleotide is affected by its neighbor, in addition to its location along the probe. Considering the second nucleotide offers more insights into the on-chip behavior of the four bases in relation to each other. Such insights are important to develop a better understanding of the on-chip hybridization process and therefore better analysis procedures. The model described here enhances the performance of an existing widely-used preprocessing algorithm for GeneChip data. We expect the same model to enhance the performance of preprocessing algorithm for other types of arrays, in particular those used for SNP analysis.

## Methods

### Datasets

#### The U133 Latin square dataset

This dataset is composed of 14 experiments (three technical replicates for each experiment) in which 42 transcripts are spiked at a concentration range of 0.125–512 pM following a Latin square design. The dataset files were downloaded from Affymetrix web site [20]. For AffyComp analysis, all probesets were included. For the 14 2× comparisons the following probesets were excluded following Affymetrix recommendations: 209374_s_at, 205397_x_at, 208010_s_at. In addition, we excluded any probesets with a name starting with AFFX- that was not included in the 42 true positive spikeins.

#### The Golden spikein dataset

This dataset has more spikein genes than the Latin Square dataset, but consists of only six microarrays, 3 C (control) and three S (spikein) [16]. The S pool contains cRNA at concentration equal to or higher than the C pool [16]. Each pool was hybridized to the Affymetrix *Drosophila *array (three technical replicates for each hybridization). Probesets measuring spikein transcripts were determined based on the analysis of [17]. We considered all probeset that measure differentially expressed genes to be true positives (a total of 1353 probesets).

Several issues have been raised concerning the use of the Golden spikein dataset in validating GeneChip preprocessing algorithms [26-28]. However, the analysis of Pearson [29] shows clearly that the Golden spikein dataset can be used to validate and compare the performance of GeneChip preprocessing algorithms.

### Model implementation

The single nucleotide model was implemented in Perl [30], the dinucleotide model was implemented in Java. All the models were fitted using the least squares method. The fitted parameters for the dinucleotide model for each of the two datasets were used to generate an affinity.info matrix for that dataset. This affinity.info matrix was used in GCRMA analysis later on. Affinity.info matrix generation was done using a local R script following the steps found in GCRMA source code (see ). The Java code for the dinucleotide model is provided at .

### Data analysis

All analysis steps were performed using R [31] version 2.5.0 and Bioconductor [32] unless otherwise indicated.

#### Expression summaries

Expression summaries were generated using the full model of GCRMA version 2.8.1. The commands used to generate the summaries for GCRMA-NN, GCRMA-L and GCRMA-R can be found at . The affinity.info matrix for the U133 Latin square dataset is provided as , and the Golden spikein dataset affinity.info matrix is provided as .

#### AffyComp analysis

Affyomp analysis was done using a locally installed AffyComp 1.14.0 package. All expression summaries were converted back from the log scale to the original scale and formatted to a comma-delimited text files using a local Perl script. Metrics generation for the expression summaries was done using a local R script following the directions of the package maintainers. The following metrics were used to evaluate the performance of each algorithm (definitions are according to Affycomp website [33]): **Median SD **is the median standard deviation across replicates. It measures the consistency of the algorithm; the lower the median SD the more consistent the algorithm. **Null log-fc IQR and null log-fc 99.9% **are the interquartile range and the 99.9^th ^percentile of the log fold changes from probesets, for genes that should not change. A perfect score is 0 for both metrics. **Signal detect slope **is the slope obtained from regressing expression values on nominal concentrations in the spikein data. **Signal detect R**^2 ^is the R squared obtained from regressing expression values on nominal concentrations in the spikein data. **Low.slope, med.slope and high.slope **are as in signal detect slope, but for probesets targeting low, medium and high spikeins, respectively. **Obs-intended-fc and Obs-(low)int-fc slopes **are slopes obtained from regressing observed log fold changes against nominal log fold changes for all probesets, and for those with nominal concentration less than 2 pM, respectively. **Low, med and high AUC **reflect the area under the ROC curve (with up to 100 false positives) for spikeins with low, medium and high intensities, standardized so that optimum is 1, respectively. **Weighted avg AUC **is the weighted average of the previous three ROC curves with weights related to amount of data in each class (low, medium and high).

#### ROC curve and cyber t analysis

ROC curve generation was implemented in Java and cyber *t *analysis was done in R. Detailed description of the implementation and the analysis can be found here [34].

## Authors' contributions

RZG participated in the design of the study, coded the single nucleotide model (equation 1) and dinucleotide model (equation 3), carried out the analysis, and drafted the manuscript. AAF implemented the single nucleotide model (equation 2) and the analysis pipeline for Cyber *t *test and ROC curve generation. AAF, RZG and CJG conceived of the study, participated in its design, coordinated the research and analysis, and drafted the manuscript.

## Supplementary Material

Additional File 1 **Boxplots showing the R^2 ^of the single nucleotide model (N) (using the 100 free parameters (N100), equation 1, and the 16 free parameters (N16), equation 2) and the dinucleotide model with 64 free parameters (NN 64), equation 4 on the 42 Latin square chips.** PM indicates the fit was done on the perfect match probes, MM indicates the fit was done on the mismatch probes, shuffled indicates the fit was done on the shuffled probe sequences and random indicates the fit was done on randomly generated probe sequences. Click here for file
