# Supplementary figures and images for: Background correction using dinucleotide affinities improves the performance of GCRMA
Source: BMC Bioinformatics. 2008 Oct 23;9:452. doi: 10.1186/1471-2105-9-452 (PMC2579310; doi:10.1186/1471-2105-9-452)

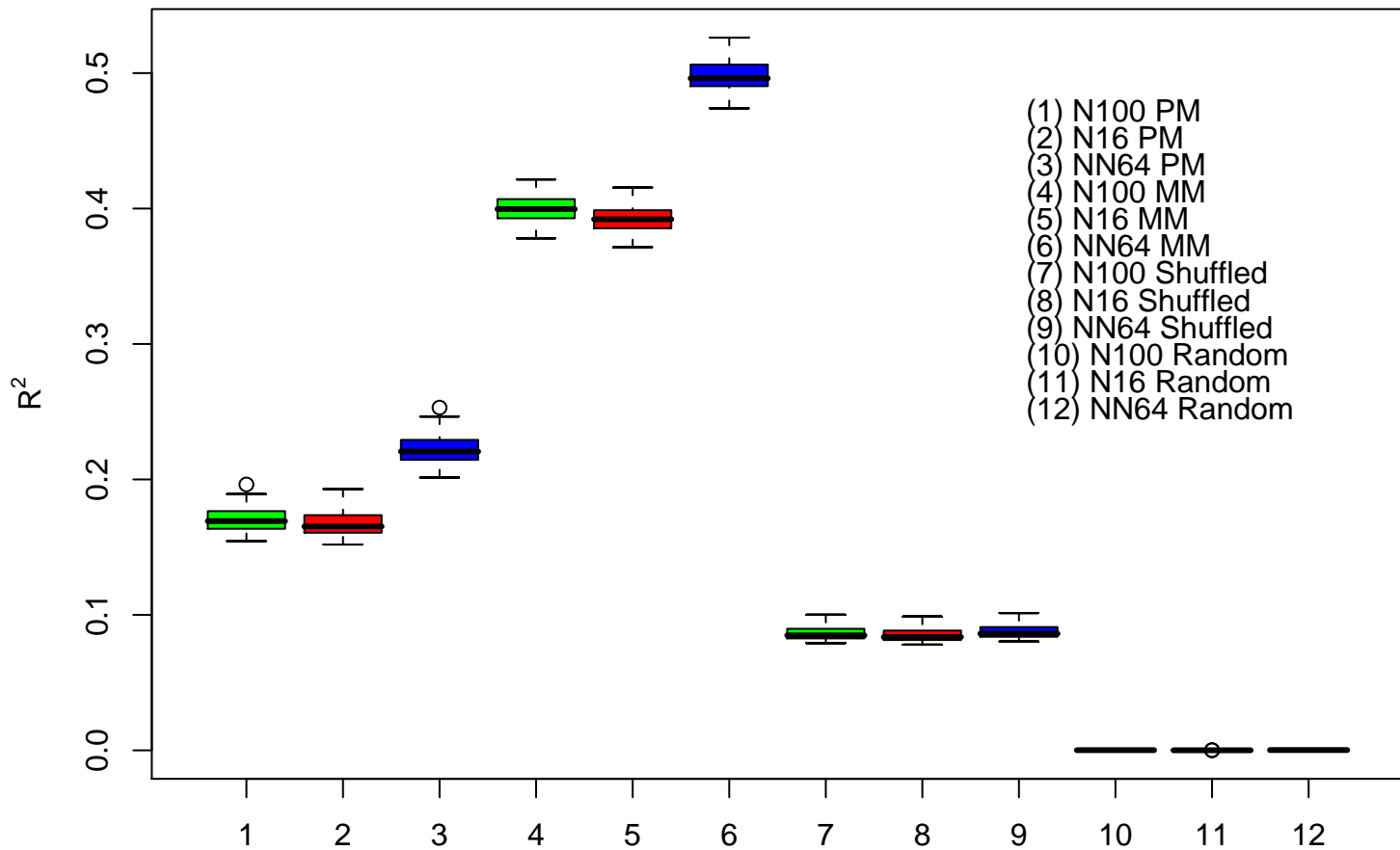

Supplement: Additional File 1 — Boxplots showing the R2 of the single nucleotide model (N) (using the 100 free parameters (N100), equation 1, and the 16 free parameters (N16), equation 2) and the dinucleotide model with 64 free parameters (NN 64), equation 4 on the 42 Latin square chips. PM indicates the fit was done on the perfect match probes, MM indicates the fit was done on the mismatch probes, shuffled indicates the fit was done on the shuffled probe sequences and random indicates the fit was done on randomly generated probe sequences. [file 1471-2105-9-452-S1.pdf]
